# A Cross-Sectional Community Readiness Assessment for Implementing School-Based Comprehensive Sexuality Education in Islamabad, Pakistan

**DOI:** 10.3390/ijerph18041497

**Published:** 2021-02-04

**Authors:** Furqan Ahmed, Ghufran Ahmad, Katharina Paff, Florence Samkange-Zeeb, Tilman Brand

**Affiliations:** 1 Department of Prevention and Evaluation, Leibniz Institute of Prevention Research and Epidemiology, 28359 Bremen, Germany; paff@leibniz-bips.de (K.P.); samkange@leibniz-bips.de (F.S.-Z.); brand@leibniz-bips.de (T.B.); 2 Health Sciences Bremen, University of Bremen, 28359 Bremen, Germany; 3 International Business & Marketing, NUST Business School (NBS), National University of Sciences and Technology (NUST), H-12 Islamabad, Pakistan; ghufran.ahmad@nbs.nust.edu.pk

**Keywords:** sexuality education, community readiness assessment, adolescent health

## Abstract

Evidence indicates that school-based sexuality education empowers children and adolescents with the skills, values, and attitudes that will enable them to appreciate their health and well-being, nourish respectful social and sexual relationships, understand their rights, and to make informed choices. Owing to organized community resistance and prevalent misconceptions, promoting sexual and reproductive health has been challenging, especially in conservative settings like Pakistan. This study aimed at systematically exploring communities’ perceptions regarding implementing school-based comprehensive sexuality education by conducting a cross-sectional community readiness assessment in Islamabad, Pakistan. A total of 35 semi-structured interviews were conducted with community key informants. Following the guidelines of the community readiness handbook, the interviews were transcribed and scored by two independent raters. The results indicate that, overall, the Islamabad community is at stage two of community readiness, the denial/resistance stage. Individual dimension scores indicate that knowledge of efforts, resources for efforts, knowledge about the issue, and leadership dimensions are at the denial/resistance stage. Only community climate was rated at stage three of community readiness, the vague awareness stage. This indicates that, for promoting sexuality education in the Pakistani context, it is essential to tackle resistance by sensitizing the community and the stakeholders through awareness campaigns.

## 1. Introduction

### 1.1. Comprehensive Sexuality Education

In 1994, the International Conference on Population and Development called for the education and promotion of adolescent sexual and reproductive health (SRH) [1]. Since then, unfortunately, progress has been gradual owing to misconceptions, organized community resistance regarding sexuality education, and implementation barriers in many regions of the world [2,3]. According to a 2014 report by the United Nations Educational, Scientific and Cultural Organization (UNESCO), there are a few examples of scaled-up intervention programs addressing sexuality education [1]. According to the UNESCO’s revised technical guidance on comprehensive sexuality education (CSE), CSE is a curriculum-based approach to teaching and learning, focusing on the cognitive, emotional, physical, and social aspects of sexuality [4]. The previous guidelines on CSE were in response to, or focused on, prevention of HIV. Research and practice, however, provided newer insights pointing to the relevance of CSE for the healthy development and overall wellbeing of children and adolescents [4]. Hence, the revised guidance expands on key concepts and includes aspects such as early pregnancy, unsafe abortion, and gender-based violence, with a special emphasis on prevention [4]. The revised technical guidance puts a greater focus on gender issues and a firm grounding in a human rights-based approach to sexuality, i.e., sexuality is a natural part of human development, which promotes structured learning in a manner that is age and developmentally appropriate [4]. The guidance also focuses on multiple Sustainable Development Goals (SDG), in particular those aimed at achieving good health and wellbeing (SDG 3), quality education (SDG 4), and gender equality (SDG 5) [4,5]. Moreover, schools provide an environment through which CSE can be implemented at an early age and sequentially over the years with a spiral approach of content building [4,6]. They also provide an active infrastructure, which includes teachers who are considered to be sources of reliable and trustworthy information by parents and offer sustainable programming opportunities [4,6]. Therefore, schools are considered to play a pivotal role in the provision of CSE, and the incremental approach is based on four age groups: 5–8 years, 8–12 years, 12–15 years, and 15–18+ years [4,6].

### 1.2. Why Sexuality Education Is Essential for Pakistan.

Pakistan is a tough setting for implementing and promoting reproductive health, women empowerment, and sexuality education [7,8]. It is taboo to talk about adolescent SRH and, as in many other countries, there is the general perception that exposure to sexuality education may lead to unwanted behaviors [7,8]. Due to a lack of formal sexuality education, adolescents resort to untrustworthy sources of information such as peers, social media, the internet, and magazines [9,10,11,12]. In addition to exposing them to harm, misinformation, mistreatment, and exploitation, this might lead to mental health problems in adolescents as indicated by a study conducted in Karachi [9,11]. Further, according to a national survey conducted in the country in 2002, only 41 percent of men and 33 percent of women had been educated and informed regarding puberty before its onset. A negative perception towards issues related to sexual organs was also observed, with almost 50% of the respondents believing that problems related to sexual organs should not be reported [13].

The World Economic Forum’s gender parity report 2020 ranked Pakistan 151st out of 153 countries [14]. Females in Pakistan have less financial independence and little to no decision-making power [15,16]. Early marriage is also prevalent and there is little acknowledgment that young girls need to be educated regarding sexuality and reproductive health rights [15,16]. A major gap that hinders progress in these aspects is the lack of SRH education in schools and its exclusion from official educational curricula [15,17]. Pakistan has a large adolescent population, and the prevention of early marriage, high fertility rates among adolescent girls, and sexual violence against children and adolescents is a challenge [18]. According to Saahil Foundation reports, 3445 cases in 2017, 3832 cases in 2018, 2846 cases in 2019, and 1489 cases in the first six months of 2020, were reported of child abuse and in almost two-thirds of the cases, the offenders were acquaintances of the victims or members of the victims’ families [19]. These numbers are compiled by monitoring multiple national and regional newspapers, but the major gap remains that there are no data registries available for monitoring and reporting the numbers of child abuse cases in Pakistan. Underreporting is also a major issue, primarily due to the associated social stigma [19]. According to the literature, strategies such as sex offender management and school-based education programs might help curtail such deplorable incidents [20], and CSE programs effectively reduce child abuse, teenage pregnancies, and sexually transmitted diseases [21].

### 1.3. Implementing CSE in Conservative Settings

According to UNESCO, “there is less clarity about how to implement (CSE) and how to scale (it) up in diverse contexts”, especially when confronted with community resistance [2]. Resistance has also been faced from parents and religious institutions in other countries, where families and parents are seen as having the right to provide sexuality education and early exposure to sexuality education are perceived to lead adolescents to engage in unwanted sexual activity [7,22,23]. The latest CSE guidelines developed by United Nations (UN) agency partners have incorporated multiple sustainable development goals (SDGs) with special emphasis on good health and well-being (SDG 3), quality education (SDG 4), and gender equality (SDG 5) [24,25]. CSE’s main aim is to empower children and adolescents with skills, values, and attitudes that will enable them to appreciate their health and well-being, nourish respectful social and sexual relationships, understand, and protect their rights, and make informed choices that affect their overall wellbeing [25,26].

### 1.4. Community Resistance to Sexuality Education in Pakistan

The literature from Pakistan suggests that a large proportion of the youth supports the introduction of SRH education [8,9,10]. The support from community influencers, decision-makers, and gatekeepers is, however, considerably feeble and a culture of silence usually persists around sexuality education [7]. School-based CSE programs have a substantial role in reaching out to large numbers with proven effectiveness and also have the added benefit of imparting age-appropriate, developmentally relevant information using a systematic, spiral approach to build on previous content and concepts regarding sexuality [24,25]. While recent publications have highlighted the importance of community engagement and of developing strategies that address the prevailing resistance to sexuality education [7,22], such upscale interventions are rare in Pakistan. Two NGOs, Aahung and Rutgers [27], tried to address the issue of resistance to school-based and out-of-school adolescent SRH interventions, by using a participatory approach to developing life skills-based content, which they tried to implement in Pakistan. At the end of the day, their efforts highlighted the central role played by community influencers as major contributors in spreading misinformation regarding the initiatives, thereby fueling organized community resistance and resulting in a backlash [7].

### 1.5. Community Readiness Model

According to findings of a literature review conducted by Stith et al. [28], the following conditions are necessary for the successful implementation of community-based health programs: a community must be ready for the preventive program, community coalitions must be developed, the program must be tailored to the community, adequate technical assistance should be available, and training/resources should be sufficiently allocated. The concept of community readiness in preventive health programs is based on increasing this readiness to have better participation and inclusion in health interventions [29,30]. Therefore, it is critical to assess community readiness and, where necessary, increase its levels through awareness and community engagement before implementing an intervention [29,30]. Several tools to assess community readiness have been developed, but the community readiness model (CRM) is the one that has widely been used in health promotion, suicide prevention, HIV/AIDS prevention, and programs aiming to improve physical activity uptake among diverse communities [30,31,32,33]. The CRM comprehensively considers the current prevention efforts, community knowledge of those efforts, leadership, community climate, knowledge about the issue, and resources for prevention [33]. The definitions of the five readiness dimensions and the respective nine readiness levels are provided in Table S1.

A considerable effort needs to be invested in the development of strategies to increase community support for CSE in Pakistan. There is a lack of research and general discussion of the kind of resistance, possible strategies to respond to resistance, and the role of influencers, leadership, and gatekeepers [7,23]. Hence, this study aimed to explore and develop an improved systematic understanding of community readiness for implementing school-based CSE in Islamabad, the capital city of Pakistan.

## 2. Materials and Methods

### 2.1. Community

Our study was based in Islamabad, Pakistan. Islamabad has a population of almost 2 million with 0.3 million households according to the 2017 census [34]. The city has a literacy rate of 82%, the highest in the country, and has 367 primary (grades 1 to 5), 162 middle (grades 6 to 8), 250 high (grades 9 and 10), and 75 higher secondary (grades 11 and 12) schools [35].

### 2.2. Tiered Approach for Assessing Community Readiness Using the Socio-Ecological Model

According to Mouli et al. [7,23], there are various levels which influence the environment of adolescents in Pakistan regarding access to sexuality education. These various levels, which correspond to the ecological framework, are categorized into five tiers, i.e., society, community, organizational, interpersonal, and individual [7]. For each tier, we categorized key respondents from Islamabad, except for the individual tier, as our focus was on gatekeepers and influencers. In each tier, respondents were recruited based on current literature regarding stakeholders that play a key role in influencing the decisions regarding implementation of health interventions, especially SRH [7,23]. A cross-sectional community readiness assessment (CRA) for implementing school-based CSE in Islamabad was conducted through key respondent interviews. The semi-structured interview guide used was adapted from the CRM handbook [33]. The questionnaire had both open-ended and closed-ended questions about the community’s attitudes to, knowledge of, and beliefs about implementing CSE in Islamabad and addressed five key dimensions; knowledge of efforts, leadership, community climate, community knowledge of the issue, and resources [33].

### 2.3. Recruitment Strategy and Data Collection

The key respondents were recruited using purposive sampling. Online searches of institutions involved in sexuality education and/or policy-making were conducted to identify potential respondents. Searches were also conducted to identify community members who might act as gatekeepers for sexuality education. Snowballing was also used to identify and recruit additional respondents. For each tier of the community, five to six key respondents were recruited, as recommended by the CRM handbook [33]. The respondents were sent an invitation to participate in the study, together with a participant information sheet and a separate fact sheet on CSE. Before the interview, informed written consent was obtained via email. During the interview, the interviewer discussed the CSE and its key features with the respondents using the fact sheet. The interviews were conducted online from April to July 2020. All interviews were recorded and then transcribed by a professional transcription company.

### 2.4. CRA Scoring System

The scoring system used was adapted from the CRM handbook [33] and is provided as Table S1. Each interview transcript was scored to give a readiness level for the five dimensions, using a nine-point rating scale; 1 (No awareness), 2 (Denial/Resistance), 3 (Vague Awareness), 4 (Preplanning), 5 (Preparation), 6 (Initiation), 7 (Stabilization), 8 (Expansion/Confirmation), and 9 (Community ownership). The interview transcripts were scored by two raters independently following the CRM scoring system (Table S1) [33]. According to the CRM handbook, the main aim of scoring the data by raters is to efficiently apply the scoring system (Table S1) to the data collected. Although, the self-reported respondent data (Table S2) provides interesting insights into community perceptions, for allocating readiness scores to dimensions only consensus scores by raters are relied upon [33].

### 2.5. Data Analysis

The reliability of the independent scores of the two raters was assessed using the intraclass correlation (ICC). ICC measures the reliability of two different raters and, therefore, is used to determine the inter-rater reliability [36]. The inter-rater reliability measures determine the robustness of the scores among raters [37]. For the estimation of the ICC, we used the two-way mixed-effects model because the same two raters scored all the interviews [38]. Moreover, the relevant estimate is based on the absolute agreement of the two raters. The ICC estimates, provided in Table S3, range from 0.42 to 0.60 for all dimensions. The inter-rater reliability, based on these estimates, can be categorized as fair according to Cicchetti & Sparrow [39], fair to good according to Fleiss [40], moderate according to Landis & Koch [41], and as good according to Regier et al. [36].

The two raters discussed and developed a consensual score for each dimension and interview as recommended by the CRM handbook [33]. The global score for the consensual score was calculated as an arithmetical mean of the five dimensions. The descriptive statistics for the consensual score—based on the dimensions, respondent category, and tiers—are provided in Table 1. A summary of the scores given by the respondents is provided in Table S2.

Regression analysis was conducted to examine the association of the respondent’s characteristics with the consensual scores for each dimension and the global score. Even though the sample size of 35 respondents is a limiting factor for regression analysis, it can still provide some interesting insights about how the respondent’s characteristics affect the scores. Since the scores were given on a nine-point rating scale, the dependent variables range from 1 to 9. Such models cannot be estimated using a linear model because the predicted values from a linear model can go beyond the range of 1–9 [42]. Additionally, the consensual scores are continuous, so ordered regressions cannot be applied. Therefore, the following transformation was used:

(1) $$Z=\frac{Y-1}{8}$$

In Equation $\left(1\right)$, the transformed variable Z lies in the interval $\left[0,\;1\right]$ if Y lies in the interval $\left[1,\;9\right]$. Hence, we rely on a fractional regression model with the logit link function, the transformation for each of the dimensions and the global score as the dependent variable. The respondent’s tier, age, duration of stay in the community, and biological sex were used as the explanatory variables. The results of the regressions are provided in Table S4. The entire analysis was conducted using Stata 14.2 (Stata Corp., College Station, TX, USA). Qualitative interview data was not analyzed as the focus of this manuscript is to analyze and interpret the community readiness scores and dimensions.

## 3. Results

### 3.1. Characteristics of Study Participants

The sociodemographic characteristics of the respondents and a summary of the duration of the interviews are provided in Table A1 [43]. 35 online interviews were conducted with 20 females and 15 males with a mean age of 31.77 years. The mean duration of the interviews was 30.78 min. Table 1 shows the number of participants in each ecological tier. Although the sample size for some of the respondent categories is small, they do offer some interesting insights. The results of the CRA are discussed in detail for each community readiness dimension hereafter:

### 3.2. Community Knowledge of Efforts

Of the 35 respondents, only 10 responded that currently there were efforts in Islamabad to address sexuality education (Table S2). On a scale of one to ten, respondents rated a mean value of 4.37 on how much of a concern sexuality education is for the community members of Islamabad (Table S2). The community’s awareness of efforts was rated from ‘none’ to ‘a few’ community members in terms of: heard of efforts, can name the efforts, know the purpose of efforts, know who the efforts are for, know how the efforts work, and the effectiveness of the efforts (Table S2). Community readiness scores for knowledge of efforts were highest for the education department representative and lowest for the religious scholar (Table A2). Overall, the global community readiness score for the dimension ‘knowledge of efforts’ was 2.03, corresponding to the denial/resistance stage of community readiness (Table 1).

### 3.3. Leadership

Almost two-thirds of the respondents believed that leadership is not supportive in expanding efforts to address sexuality education in Islamabad (Table S2). On a scale of one to ten, respondents rated the level of concern regarding sexuality education for the leadership in Islamabad with a mean value of 3.66 (Table S2). Looking at the leadership dimension, the health department representative gave the highest score and the community leader and head of schools the lowest (Table A2). The overall global score for the leadership dimension was 2.50, corresponding to the denial/resistance stage of community readiness (Table 1).

### 3.4. Community Climate

When asked to rate how much of a priority sexuality education is for community members in Islamabad, on a scale of one to ten, a mean value of 2.89 was given by the respondents (Table S2). The respondents further believed that many community members at least passively support community efforts without being actively involved in any way and that a few played key roles as leaders or driving forces in implementing efforts (Table S2). Regarding the willingness of community members to pay more in terms of taxes to help fund community efforts regarding sexuality education, the respondents generally believed that none to a few would be willing to do so (Table S2). They also thought that only a few community members would support expanding efforts regarding sexuality education in Islamabad (Table S2). For this dimension, the highest readiness score was given by political activists and the lowest by teachers from rural Islamabad (Table A2). The overall global score for the community climate dimension was 3.37, corresponding to the vague awareness stage of community readiness.

### 3.5. Knowledge about the Issue

On a scale of one to ten, the level of community members’ knowledge about sex education in Islamabad was given a mean value of 3.97 (Table S2). The respondents were also of the opinion that community members know nothing to a little about the topics and contents of CSE, benefits of school-based adolescent health interventions, issues regarding adolescent health in the community, how adolescent health can be improved, and information regarding the age-appropriateness of sexuality education (Table S2). The highest readiness score for this dimension was given by the education department representative and the lowest by NGO representatives (Table A2). The overall global readiness score for knowledge about the issue dimension was 2.06, corresponding to the denial/resistance stage of community readiness (Table 1).

### 3.6. Resources for Efforts

Regarding resources for efforts, the respondents thought that there were very few volunteers and little space for conducting activities on sexuality education in Islamabad (Table S2). They thought that resources in terms of financial donations, grant funding, and experts’ availability for addressing sexuality education in Islamabad were nonexistent to just a little (Table S2). The respondents also believed that, currently, no efforts were being made to seek volunteers, solicit donations for expanding efforts, write grant proposals, train community members to become experts, or recruit experts in Islamabad (Table S2). The highest readiness score for this dimension was observed for community leaders and the lowest for social media influencers and the education department (Table A2). The global score for the resources for efforts dimension was 2.89, which corresponds to the denial/resistance stage of community readiness (Table 1).

### 3.7. Regression Analysis

The results of the regression analysis are provided in Table S4. Regarding the respondents’ tier and biological sex, the community tier and female were taken as the base in the regression. The following inferences are drawn for coefficients that are significant at the 5% level. The results show that the respondents belonging to the interpersonal tier (parents) gave a lower score on knowledge of efforts, leadership, resources for efforts, and global score, compared to the respondents in the community tier. On the other hand, respondents in the organizational tier gave a higher global readiness score compared to those in the community tier. Moreover, respondents in the society tier were more optimistic regarding leadership and global scores than respondents in the community tier. In general, older respondents were more positive regarding knowledge of efforts and more pessimistic about resources, regardless of their tier. Respondents who had lived in the community for a longer duration were more confident about the community climate and resources. In terms of biological sex, male respondents were more doubtful about the knowledge of issue dimension.

## 4. Discussion

This study aimed to systematically investigate the level of community readiness regarding the implementation of school-based CSE in Islamabad. Overall, the results of this study highlight that community readiness currently corresponds to the denial/resistance stage, which is not conducive to the effective promotion, development, and integration of CSE in the school-based curriculum in Islamabad. The low readiness levels observed were attributed to misconceptions, lack of awareness, community resistance towards efforts, lack or absence of leadership support, and scarcity of resources dedicated to such efforts.

### 4.1. Lack of Awareness

The denial/resistance stage of community readiness suggests that some members of the community recognize the issue under discussion but, due to lack of awareness and the prevalence of misconceptions in society, do not recognize its entire relevance [33]. The CRM handbook suggests that interventions can be utilized to raise the readiness levels of communities at stage two of readiness, before the implementation of health promotion interventions [33]. According to Mouli et al. [7], collaborating with leaders to identify priority issues, carefully considering sensitivities, tactfully framing issues, engaging community influencers, and improving media presence are some of the strategies that can be applied to tackle resistance to CSE in conservative settings such as Pakistan. The authors also recommend that, when it comes to interventions involving controversial or taboo topics, the reasons for the low levels of community readiness should be investigated, and the interventions should be tailored with careful stakeholder involvement from the community so that the intervention is not dismissed outright.

The fact that less than a third of the 35 respondents were aware of any community efforts addressing sexuality education in Islamabad and that the lowest readiness score across all the dimensions was observed for community knowledge of efforts, might be due to the associated stigma, that leads to a lack of dissemination of information regarding these efforts to the public [7]. The lowest score for knowledge of efforts also resonates with the respondents’ perceptions that sexuality education is not a concern and a priority for the leadership in Islamabad. However, in recent years, the Ministry of National Health Services, Regulation, and Coordination of Pakistan has initiated a lot of work on sexual and reproductive health [44]. In collaboration with international organizations, programs incorporating leadership support on SRH, focusing on service delivery and utilization, are underway. This probably explains the fact that, of all categories of respondents, health department representatives assigned the leadership dimension the highest score. Nevertheless, no efforts are being made to integrate CSE in the school-based curriculum at the moment.

### 4.2. Community Support and Recent Developments

Although the global community climate was scored the highest among all dimensions, the score corresponds only to vague awareness among community members. The respondents’ perception that sexuality education is of low priority for community members in Islamabad and that community members might passively support efforts in this regard rather than being actively involved in developing and implementing such efforts resonates with the general stigma and resistance in the Pakistani community towards such efforts [7]. Resistance is further mirrored in the low priority community members reported for the expansion of efforts regarding sexuality education and the perception that not many would be willing to pay more for this. Recent concerns among the community regarding violence against women and children have been flagged by community efforts such as “Aurat (Woman) March” and online platforms by influencers who have been successful in initiating discussions, usually passive, on online platforms [45,46]. A recent case of the rape and murder of a six year old in Kasur, Punjab, the “Zainab case”, has brought such issues to National level platforms which have led to an increase in support from the community and leadership to develop legislation on protection of children and women in the form of the “Zainab Bill” [47,48]. Although such events create momentary efforts, they fail to translate the momentum into substantial continuous organized endeavors such as integrating sexuality education concepts into the school-based curriculum. These efforts are directed towards mitigating such unfortunate events and do not entail the initiation of long-standing efforts towards significant actions culminating in the promotion and preventive measures at the community level.

### 4.3. Lack of Knowledge Concerning CSE

Community members’ reported a lack of knowledge about CSE, the benefits of school-based adolescent health interventions, issues regarding adolescent health in the community, how adolescent health can be improved, and information regarding age appropriateness of sexuality education. These are some of the underlying reasons that nurture community resistance. Although there is evidence highlighting the potential of CSE to improve adolescent health and at the same time empower adolescents to make healthy and right choices as they grow older and develop [24,25], there is a concern and misconception among parents that the content of CSE might lead to unwanted consequences and is not age-appropriate or culturally sensitive [7]. Although there are not many upscale examples from Pakistan where CSE has been developed in the form of a curriculum, according to the UNESCO guidelines on developing CSE curriculum the main emphasis is on a stepwise age-appropriate approach [24,25]. Before the interviews, when the respondents were given a brief introduction to CSE highlighting the age-appropriateness of the contents, most of the respondents were surprised that age-appropriate content can be developed for adolescents. This further underlines the fact that lack of awareness is a key contributor that strengthens misconceptions and organized community resistance.

### 4.4. Low Support and Priority for Resources

The low priority given to sexuality education in the community and by the leadership is further reflected in the low score for the resources dimension. Lack of resources is one of the key factors that make the initiation of efforts and programs a major challenge. There is a need for increased awareness and priority building measures to garner community and leadership commitment to work towards developing and integrating sexuality education in the curriculum. Rigorous teacher training to deliver content on sexuality education has also been emphasized as an integral part of successfully integrating CSE into the curriculum [49,50,51]. In a study conducted in sub-Saharan Africa, one of the major barriers identified was the reluctance of teachers to teach sexuality education content [49,50,51]. The authors argued that the discomfort of teachers may nurture the perception of students that sexuality-related topics are taboo. One of the reasons for this hesitation in teaching CSE was probably related to the lack of training and skills to efficiently introduce sexuality-related concepts to children, as poor or no teacher training is a common hurdle when implementing CSE programs in low- and middle-income countries [49,50,51].

### 4.5. Implementation Approaches

UNESCO’s international technical guidance on CSE stresses that CSE programs should be tailored and optimized in the local context, culturally and religiously, for their uptake and implementation to be successful [24,25]. In recent studies, stakeholder engagement at the policy and community levels has also been stressed as being integral for sustaining CSE in national-level programs [7,50]. In their review of large-scale sexuality education programs successfully implemented in Pakistan, Chandra-Mouli et al. discussed two major approaches that were considered and implemented by NGOs; human rights-based and conservative religious-based [7,52]. While the conservative religious-based approach might be able to tackle resistance, the accuracy of the content and syllabus might be debatable, as it is challenging to ensure the accuracy of content and context of the sexuality education syllabus following the conservative approach [7]. Moreover, an upscale study from Pakistan also included in the review highlighted that the syllabus lacked detail in the content on sexual diversity, contraception, and abortion, which usually is the case while taking a religious approach to sexuality education [7,52]. Further, the revised UNESCO guidelines also put stress on human rights and consider the concept of sexuality as a natural part of human development centered on the best interest of adolescents [25].

### 4.6. Limitations and Strengths

Although the community readiness model offers a systematic methodology to appraise the level of a community’s readiness regarding a specific issue, it also entails some limitations. CRM is a subjective methodology that can be used to conduct a cross-sectional assessment of community readiness [33]. According to the authors of the CRM handbook, the fact that the community and the issue change with every application of the tool limit the assessment of the model’s scientific validity. They refer to the assessment tool as “Broad Scale Theory”, implying that it incorporates multiple phenomena in terms of opinions and facts, and tries to establish the possible relationships existing among the phenomena. As the CRM relies on detailed interviews with a small sample of respondents in a geographically specific community, the results cannot be generalized to other communities in terms of readiness scores [33]. However, the model has been successfully adopted to address a vast range of health, social, and environmental issues in different geographical settings, which adds to its credibility and validity [33]. The results of this study highlight potentially important issues concerning community readiness and the implementation of sexuality education in Islamabad. Incorporating the socio-ecological model in the community readiness assessment proved to be beneficial as we were able to recruit respondents from different tiers of the community. The tiered approach incorporated the perceptions of different community influencers and gatekeepers, presenting a holistic picture of the communities’ current stages of readiness. Additionally, the organized approach of the CRM assessment tool made the process of determining the readiness stage for different dimensions efficient and provided the respondents with the opportunity to give their perspectives about their communities.

## 5. Conclusions

School-level implementation barriers are only a small part of the challenges that hinder the implementation of CSE programs. Other barriers, for instance, policy level planning, leadership engagement, community mobilization, and support, and resource allocation also need to be addressed. For the successful implementation of CSE, there must be an enabling environment, which includes support and investment at the policy and community levels coupled with infrastructural backing. As of today, the community readiness level for the successful implementation of CSE in Islamabad was found to be low. Efforts should be directed at creating awareness, garnering support, and prioritizing CSE within different tiers of the community.

## Figures and Tables

**Table 1:** Community readiness dimension score, respondent category readiness scores, and tier wise readiness scores based on rater consensus scores.

**Community Readiness Dimension Score Descriptive Statistics**			
**Community Readiness Dimensions**		**Mean**	Std. Dev. *
Knowledge of Efforts		2.03	1.27
Leadership		2.50	0.99
Community Climate		3.37	0.69
Knowledge of issue		2.06	0.54
Resources		2.89	0.88
Global Score		2.57	0.55
**Respondent Category Readiness Scores and Descriptive Statistics**			
**Socio Ecological Tier**	**Respondents**	**Mean**	Std. Dev. *
Interpersonal Mean = 2.27 Std. Dev. * = 0.35	Parent (n = 6)	2.27	0.35
Organizational Mean = 2.59 Std. Dev. * = 0.69	NGO (n = 5)	2.68	1.01
	Teacher/Rural (n = 4)	2.48	0.42
	Teacher/Urban (n = 4)	2.83	0.64
	Head of School (n = 2)	2.15	0.21
Community Mean = 2.51 Std. Dev. * = 0.37	Social Media Influencer (n = 2)	2.35	0.21
	News channel (n = 1)	2.30	NA **
	Doctor (n = 2)	2.45	0.21
	Religious Scholar (n = 1)	2.40	NA **
	Community Leader (n = 1)	3.30	NA **
Society Mean = 2.83 Std. Dev. * = 0.44	Education Department (n = 1)	3.20	NA **
	Political activist (n = 2)	2.80	0.28
	Health Department (n = 4)	2.75	0.56

* Standard deviation, ** Only one observation hence calculating standard deviation not possible.

## Data Availability

The data presented in this study are available from the corresponding author on reasonable request.

## Supplementary Materials

The following are available online at https://www.mdpi.com/1660-4601/18/4/1497/s1, Table S1: CRA Scoring System, Table S2: Descriptive statistics on five dimensions of community readiness based on respondent self-reported data Table S3: Inter-rater reliability, measured by the intraclass correlation coefficient, for independent rater interview scoring, Table S4: Regression analysis for five community readiness dimensions and global scores.

## Appendix A

**Table A1:** Key respondents’ details, Participant, and Interview Descriptive Statistics.

Variable	Mean	Median	
Interview Duration	30.78	29.51	
Age	31.77	30.00	
How long have you lived in the community	23.91	24.00	
**Variable**		**Frequency**	**Percent**
Sex	Male	15	42.85
	Female	20	57.14
Do you live in the community?	No	1.00	2.86
	Yes	34.00	97.14
Do you work in the community?	No	1.00	2.86
	Yes	34.00	97.14

**Table A2:** Descriptive statistics of community readiness dimensions by respondent category.

Respondent Category	Knowledge of Efforts		Leadership	Community Climate			Knowledge of Issue		Resources	
	Mean	Std. Dev. *	Mean	Std. Dev. *	Mean	Std. Dev. *	Mean	Std. Dev. *	Mean	Std. Dev. *
Community Leader	4.50	NA	1.00	NA	3.50	NA	2.50	NA	5.00	NA
Doctor	2.00	0.00	2.50	0.71	3.25	0.35	2.00	0.00	2.50	0.71
Education Department	5.00	NA	3.00	NA	3.00	NA	3.00	NA	2.00	NA
Head of School	1.50	0.71	1.00	0.00	3.25	1.06	2.50	0.71	2.50	0.71
Health Department	1.75	0.87	3.75	1.26	3.13	0.85	2.00	0.00	3.13	0.25
NGO	1.80	0.84	2.50	1.22	4.00	0.71	1.90	1.02	3.20	1.79
News channel	1.00		2.00		3.50		2.00		3.00	
Parent	1.25	0.42	2.00	0.63	3.25	0.76	2.08	0.66	2.75	0.42
Political activist	2.75	2.47	2.50	0.71	3.75	0.35	2.00	0.00	3.00	0.00
Religious Scholar	1.00	NA	3.00	NA	3.00	NA	2.00	NA	3.00	NA
Social Media Influencer	1.50	0.71	2.75	0.35	3.50	0.71	2.00	0.00	2.00	0.00
Teacher/Rural	2.25	1.44	2.63	0.48	2.75	0.87	1.75	0.50	3.00	0.82
Teacher/Urban	2.88	1.75	2.75	0.96	3.63	0.25	2.13	0.25	2.75	0.50

* Standard deviation.
