# Supplementary material for: A Cross-Sectional Community Readiness Assessment for Implementing School-Based Comprehensive Sexuality Education in Islamabad, Pakistan
Source: Int J Environ Res Public Health. 2021 Feb 4;18(4):1497. doi: 10.3390/ijerph18041497 (PMC7914735; doi:10.3390/ijerph18041497)
Supplement: Supplementary file 1 [file ijerph-18-01497-s001.zip › Table S1.docx]

Table S1: CRA Scoring System

| Stage | Knowledge of Efforts | Leadership | Community Climate | Knowledge of issue | Resources |
| --- | --- | --- | --- | --- | --- |
| 1 (No awareness)  Cut off range $\left[ \boldsymbol{1} \right.\boldsymbol{,}\left. \boldsymbol{2} \right)$ | Community members have no knowledge about local efforts addressing the issue. | Leadership believes that the issue is not a concern | Community members believe that the issue is not a concern | Community members have no knowledge about the issue | There are no resources available for (further) efforts |
| 2 (Denial/resistance)  Cut off range $\left[ \boldsymbol{2} \right.\boldsymbol{,}\left. \boldsymbol{3} \right)$ | Only a few community members have any knowledge about local efforts addressing the issue. Community members may have misconceptions or incorrect knowledge about local efforts (e.g. their purpose or who they are for) | Leadership believes that this issue may be a concern in this community, but doesn’t think it can or should be addressed | Community members believe that this issue may be a concern in this community, but don’t think it can or should be addressed | Only a few community members have any knowledge about the issue.  Among many community members, there are misconceptions about the issue Among many community members, there are misconceptions about the issue | There are very limited resources (such as one community room) available that could be used for further efforts. There is no action to allocate these resources to this issue. Funding for any current efforts is not stable or continuing |
| 3 (Vague awareness)  Cut off range $\left[ \boldsymbol{3} \right.\boldsymbol{,}\left. \boldsymbol{4} \right)$ | At least some community members have heard of local efforts, but little else | At least some of the leadership believes that this issue may be a concern in this community. It may not be seen as a priority. They show no immediate motivation to act | Some community members believe that this issue may be a concern in the community, but it is not seen as a priority. They show no motivation to act | At least some community members have heard of the issue, but little else. Among some community members, there may be misconceptions about the issue. Community members may be somewhat aware that the issue occurs locally | There are some resources (such as a community room, volunteers, local professionals, or grant funding or other financial sources) that could be used for further efforts. There is little or no action to allocate these resources to this issue |
| 4 (Preplanning)  Cut off range $\left[ \boldsymbol{4} \right.\boldsymbol{,}\left. \boldsymbol{5} \right)$ | At least some community members have heard of local efforts and are familiar with the purpose of the efforts | At least some of the leadership believes that this issue is a concern in the community and that some type of effort is needed to address it. Although some may be at least passively supportive of current efforts, only a few may be participating in developing, improving or implementing efforts | Some community members believe that this issue is a concern in the community and that some type of effort is needed to address it. Although some may be at least passively supportive of efforts, only a few may be participating in developing, improving or implementing efforts | At least some community members know a little about causes, consequences, signs and symptoms. At least some community members are aware that the issue occurs locally | There are some resources identified that could be used for further efforts. Some community members or leaders have looked into or are looking into using these resources to address the issue |
| 5 (Preparation)  Cut off range $\left[ \boldsymbol{5} \right.\boldsymbol{,}\left. \boldsymbol{6} \right)$ | At least some community members have heard of local efforts, are familiar with the purpose of the efforts, who the efforts are for, and how the efforts work | At least some of the leadership is participating in developing, improving, or implementing efforts, possibly being a member of a group that is working toward these efforts or being supportive of allocating resources to these efforts | At least some community members are participating in developing, improving, or implementing efforts, possibly attending group meetings that are working toward these efforts | At least some community members know some about causes, consequences, signs and symptoms. At least some community members are aware that the issue occurs locally | There are some resources identified that could be used for further efforts to address the issue. Some community members or leaders are actively working to secure these resources; for example, they may be soliciting donations, writing grant proposals, or seeking volunteers |
| 6 (Initiation)  Cut off range $\left[ \boldsymbol{6} \right.\boldsymbol{,}\left. \boldsymbol{7} \right)$ | Many community members have heard of local efforts and are familiar with the purpose of the effort. At least some community members know who the efforts are for and how the efforts work | At least some of the leadership plays a key role in participating in current efforts and in developing, improving, and/or implementing efforts, possibly in leading groups or speaking out publicly in favor of the efforts, and/or as other types of driving forces | At least some community members play a key role in developing, improving, and/or implementing efforts, possibly being members of groups or speaking out publicly in favor of efforts, and/or as other types of driving forces | At least some community members know some about causes, consequences, signs and symptoms.  At least some community members have some knowledge about how much it occurs locally and its effect on the community | New resources have been obtained and/or allocated to support further efforts to address this issue |
| 7 (Stabilization)  Cut off range $\left[ \boldsymbol{7} \right.\boldsymbol{,}\left. \boldsymbol{8} \right)$ | Many community members have heard of local efforts, are familiar with the purpose of the effort, who the efforts are for, and how the efforts work. At least a few community members know the effectiveness of local efforts | At least some of the leadership plays a key role in ensuring or improving the long- term viability of the efforts to address this issue, for example by allocating long-term funding | At least some community members play a key role in ensuring or improving the long- term viability of efforts (e.g., example: supporting a tax increase). The attitude in the community is ―We have taken responsibility | At least some community members know a lot about causes, consequences, signs and symptoms.  At least some community members have some knowledge about how much it occurs locally and its effect on the community | A considerable part of allocated resources for efforts are from sources that are expected to provide stable or continuing support |
| 8 (Expansion/confirmation)  Cut off range $\left[ \boldsymbol{8} \right.\boldsymbol{,}\left. \boldsymbol{9} \right)$ | Most community members have heard of local efforts and are familiar with the purpose of the effort. Many community members know who the efforts are for and how the efforts work. Some community members know the effectiveness of local efforts | At least some of the leadership plays a key role in expanding and improving efforts, through evaluating and modifying efforts, seeking new resources, and/or helping develop and implement new efforts | The majority of the community strongly supports efforts or the need for efforts. Participation level is high.  ―We need to continue our efforts and make sure what we are doing is effective | Most community members know a lot about causes, consequences, signs and symptoms.  At least some community members have a lot of knowledge about how much it occurs locally, its effect on the community, and how to address it locally | A considerable part of allocated resources for efforts are from sources that are expected to provide continuous support. Community members are looking into additional support to implement new efforts |
| 9 (Community ownership)  Cut off vale $\boldsymbol{= 9}$ | Most community members have extensive knowledge about local efforts, knowing the purpose, who the efforts are for and how the efforts work. Many community members know the effectiveness of the local efforts | At least some of the leadership is continually reviewing evaluation results of the efforts and is modifying financial support accordingly | The majority of the community is highly supportive of efforts to address the issue.  Community members demand accountability | Most community members have detailed knowledge about the issue, knowing detailed information about causes, consequences, signs and symptoms. Most community members have detailed knowledge about how much it occurs locally, its effect on the community, and how to address it locally | Diversified resources and funds are secured, and efforts are expected to be ongoing.  There is additional support for new efforts |
