# Supplementary material for: A Cross-Sectional Community Readiness Assessment for Implementing School-Based Comprehensive Sexuality Education in Islamabad, Pakistan
Source: Int J Environ Res Public Health. 2021 Feb 4;18(4):1497. doi: 10.3390/ijerph18041497 (PMC7914735; doi:10.3390/ijerph18041497)
Supplement: Supplementary file 1 [file ijerph-18-01497-s001.zip › Table S2.docx]

| Statistic | Knowledge of Efforts | Leadership | Community Climate | Knowledge of Issue | Resources | Global |
| --- | --- | --- | --- | --- | --- | --- |
| ICC | 0.48 | 0.59 | 0.50 | 0.42 | 0.53 | 0.60 |

Table S2: Inter rater reliability, measured by intraclass correlation coefficient, for independent rater interview scoring
