# Supplementary material for: A Cross-Sectional Community Readiness Assessment for Implementing School-Based Comprehensive Sexuality Education in Islamabad, Pakistan
Source: Int J Environ Res Public Health. 2021 Feb 4;18(4):1497. doi: 10.3390/ijerph18041497 (PMC7914735; doi:10.3390/ijerph18041497)
Supplement: Supplementary file 1 [file ijerph-18-01497-s001.zip › Table S3.docx]

Table S3: Regression analysis for five community readiness dimensions and global scores

|  | (1) | (2) | (3) | (4) | (5) | (6) |
| --- | --- | --- | --- | --- | --- | --- |
| VARIABLES | Knowledge of Efforts | Leadership | Community Climate | Knowledge of issue | Resources | Global |
| **Tier (ref. Community)** | | | | | | |
| Interpersonal | -1.49* | -0.37 | -0.05 | 0.01 | -0.13 | -0.23 |
|  | (0.85) | (0.36) | (0.21) | (0.26) | (0.29) | (0.15) |
| Organizational | 0.17 | -0.14 | 0.15 | -0.01 | 0.09 | 0.07 |
|  | (0.50) | (0.29) | (0.15) | (0.20) | (0.32) | (0.16) |
| Society | 0.14 | 0.62** | 0.01 | 0.02 | 0.12 | 0.19 |
|  | (0.84) | (0.30) | (0.19) | (0.13) | (0.30) | (0.16) |
|  |  |  |  |  |  |  |
| Age | 0.04* | -0.01 | 0.00 | 0.01 | -0.02** | 0.01 |
|  | (0.02) | (0.01) | (0.01) | (0.01) | (0.01) | (0.01) |
| Duration Lived | 0.00 | -0.02 | 0.01 | 0.02 | 0.02* | 0.01 |
|  | (0.02) | (0.01) | (0.01) | (0.01) | (0.01) | (0.01) |
| **Sex (ref. Female)** | | | | | | |
| Male | -0.95 | -0.00 | 0.07 | -0.24 | -0.01 | -0.13 |
|  | (0.59) | (0.26) | (0.14) | (0.18) | (0.24) | (0.14) |
|  |  |  |  |  |  |  |
| Constant | -2.88*** | -0.81* | -1.21*** | -2.60*** | -1.20*** | -1.68*** |
|  | (0.74) | (0.45) | (0.31) | (0.42) | (0.36) | (0.24) |
| Observations | 35 | 35 | 35 | 35 | 35 | 35 |
| R-squared | 0.27 | 0.28 | 0.07 | 0.17 | 0.10 | 0.14 |
| Robust standard errors in parentheses  *** p<0.01, ** p<0.05, * p<0.1 | | | | | | |
