# Supplementary figures and images for: A Cross-Sectional Community Readiness Assessment for Implementing School-Based Comprehensive Sexuality Education in Islamabad, Pakistan
Source: Int J Environ Res Public Health. 2021 Feb 4;18(4):1497. doi: 10.3390/ijerph18041497 (PMC7914735; doi:10.3390/ijerph18041497)

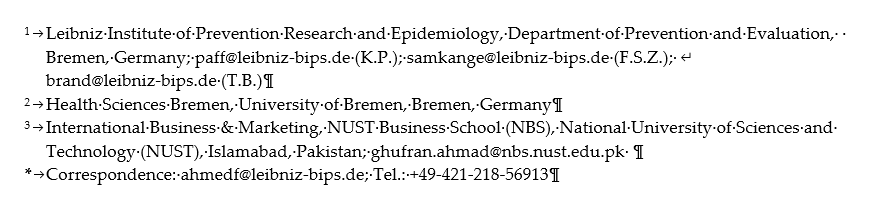

Supplement: Supplementary file 1 [file ijerph-18-01497-s001.zip › 1.png]
